# A record of changes in the Gran Sasso groundwater before, during and after the 2016 Amatrice earthquake, central Italy

**DOI:** 10.1038/s41598-018-34444-1

**Published:** 2018-10-29

**Authors:** Gaetano De Luca, Giuseppe Di Carlo, Marco Tallini

**Affiliations:** 1 Istituto Nazionale di Geofisica e Vulcanologia – Osservatorio Nazionale Terremoti, L’Aquila, Italy; 2 Istituto Nazionale di Fisica Nucleare – Laboratori Nazionali del Gran Sasso, L’Aquila, Italy; 3 Università degli Studi dell’Aquila – Dipartimento di Ingegneria Civile, Edile-Architettura e Ambientale, L’Aquila, Italy

## Abstract

We performed continuous recordings (May 2015 – January 2017) of hydraulic pressure and electrical conductivity of groundwater in the 190 m-long horizontal S13 borehole drilled next to the deep underground laboratories of Gran Sasso (*LNGS-INFN*), located in the core of the Gran Sasso carbonate aquifer (central Italy) at a distance of about 39 km south-eastward from the 24 August 2016 Amatrice earthquake (6.0 M_w_) epicenter. Using a 3-channel, 24-bit ADC we achieved a sampling rate of groundwater physical properties up to 50 Hz for each channel. We focused on the analysis of data recorded before, during and after the Amatrice earthquake, describing and discussing in detail the evidence for significant hydraulic pressure and electrical conductivity anomalies recorded before the main shock. We identified unambiguous signals in the hydraulic pressure data starting on 19 August, i.e. five days before the 24 August mainshock. A more careful analysis allowed us to detect the inception of a weak change up to 40 days before the Amatrice earthquake and a significant variation in the electrical conductivity data about 60 days before. The data revealed highly dynamic aquifer behaviour associated with the uprising of geogas probably related to the preparation stage of the Amatrice earthquake.

## Introduction

Earthquake prediction is a widely recognized goal as well as a challenging scientific problem because of its complexity and its social impact. It is easy to find many papers, reviews, debates, discussions and comments in the scientific literature^1–8^. Chemical and physical groundwater parameters have been monitored in seismogenic areas worldwide, principally by measuring and analysing water level and hydrochemistry changes in wells, springs and streams to find possible correlations with local and regional seismicity and between their spatial and temporal variations and strain processes^9–28^. Moreover, in the past few decades many studies have suggested a connection between fault mechanics and underground fluid dynamics, and a large quantity of experimental results and models have appeared in the literature^29–37^. Among these studies, those concerning the fault frictional properties in the presence of fluid can provide unique insights into fluid-rock interactions^34^.

**Figure 1:**
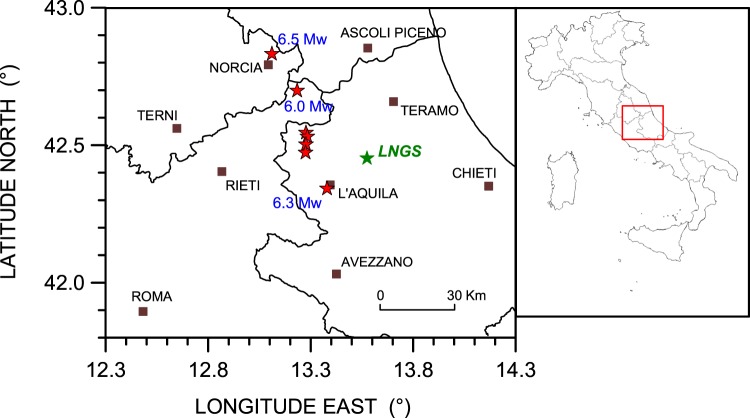
Main earthquakes location map, from Iside data-base^53^, of August 2016–January 2017 period (http://cnt.rm.ingv.it/iside); brown squares represent principal towns in the area, black lines for regional boundaries, green star for *LNGS* (Laboratori Nazionali del Gran Sasso) of *INFN* (Istituto Nazionale di Fisica Nucleare) close to the S13 horizontal borehole (distance about 200 m); from northern: red stars for 6.5 M_w_ of 30 October 2016, 6.0 M_w_ of 24 August 2016, 5.1 M_w_ of 18 January 2017 (09:25:40 UT), 5.5 M_w_ of 18 January 2017 (10:14:10 UT), 5.4 M_w_ of 18 January 2017 (10:25:24 UT) and 5.0 M_w_ of 18 January 2017 (13:33:37 UT). We also show the L’Aquila earthquake of 6 April 2009 (6.3 M_w_ – 01:32:40 UT).

In this framework, we decided to monitor some physical parameters of the deep groundwater of the large Gran Sasso carbonate aquifer (Abruzzi region, central Apennines, Fig. 1) through a horizontal borehole (named S13) placed in the highway tunnel very close to the underground laboratories of Gran Sasso (*LNGS-INFN*). The Gran Sasso chain is placed in a seismically active area of central Apennines, as demonstrated by the 6 April 2009, M_w_ 6.3 L’Aquila earthquake^38,39^ and by the recent 24 August 2016, M_w_ 6.0 Amatrice earthquake^40,41^ (Fig. 1), and provides a unique opportunity to study an inner, unexploited portion of a very large carbonate aquifer (about 1,000 km^2^). This area of the central Apennines is monitored by a rather dense regional seismic network^42^ along with the national seismic network of *INGV-ONT (*Istituto Nazionale di Geofisica e Vulcanologia – Osservatorio Nazionale Terremoti)^43^.

Convincing evidence exists in the Gran Sasso chain and its neighbouring areas that high fluid pressure contributed to the rupture during the M_w_ 6.3 L’Aquila earthquake of 6 April 2009^44–47^. Temporal variations of seismic velocity, anisotropy, slow-slip and ground deformation were also observed before the main event^48–50^. Specifically, some investigators observed clear variations in the seismic wave propagation properties about a week before the L’Aquila mainshock and inferred that fluids played a key role in the fault failure process^44–48^. Similarly, during the 24 August 2016, M_w_ 6.0 Amatrice earthquake there was evidence of hydrogeochemical and hydrogeological changes before and during the seismic sequence^51,52^, that included about 80,000 events^53^. Several geochemical anomalies were interpreted as reliable seismic precursors for an extensional tectonic setting^51^.

**Figure 2:**
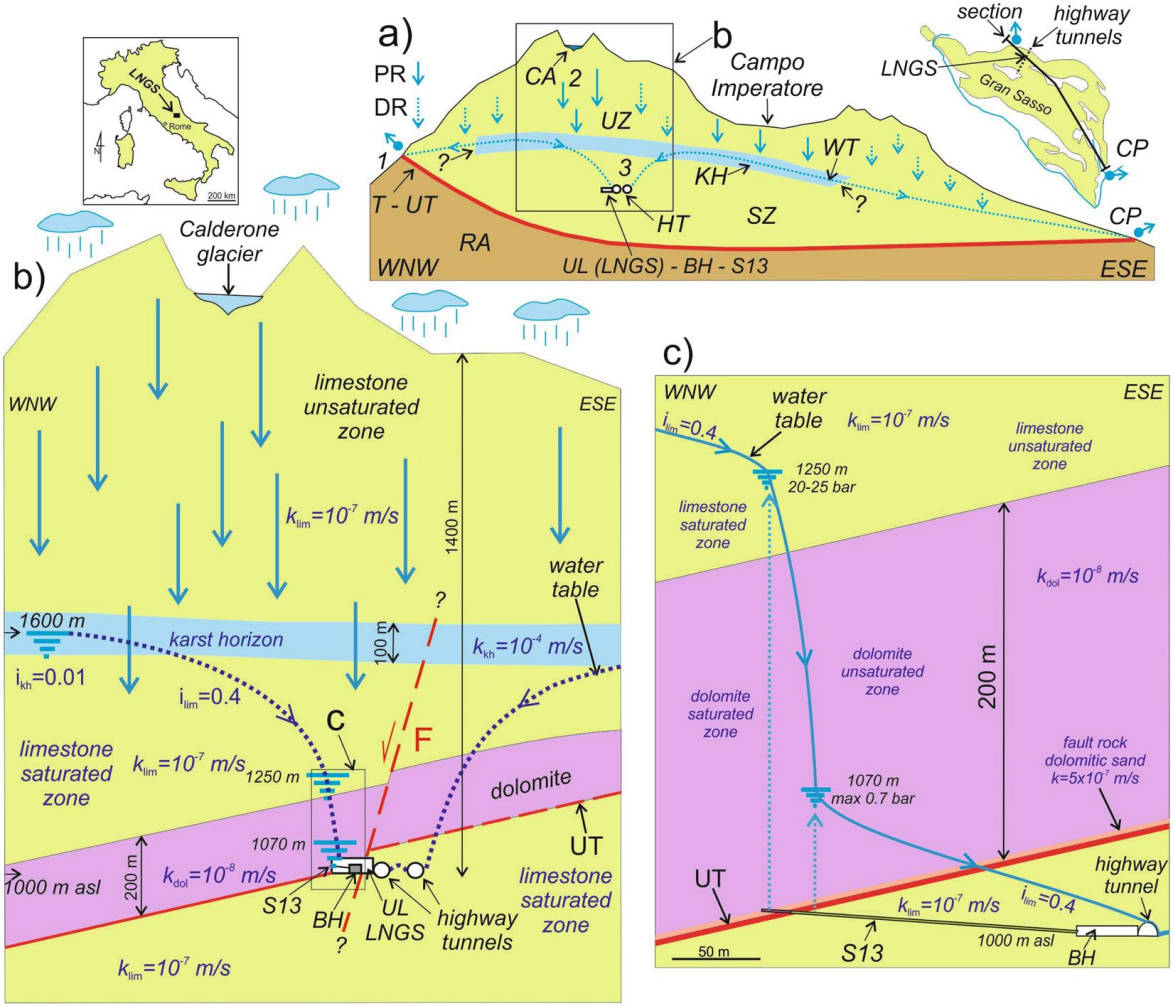
(**a**) Scheme (not in scale) of the Gran Sasso aquifer transversal to the highway tunnels and passing through the underground laboratories of Gran Sasso (*LNGS)*, borehole hall and S13 area^77^. UZ: Unsaturated Zone; SZ: Saturated Zone; KH: Karst Horizon; RA: Regional Aquiclude; T: permeability boundary (regional Thrust); UT: local thrust named Upper Thrust; WT: Water Table; HT: Highway Tunnels; UL LNGS: Underground Laboratories; BH: Borehole Hall; CA: Calderone glacier (high elevation water reservoir – preferential recharge area); 1: overflow spring (CP: Capo Pescara spring); 2: preferential groundwater flowpath area; 3: preferential groundwater flowing toward the UL; PR: Preferential Recharge; DR: Diffuse Recharge; S13: monitored horizontal borehole. The hydrogeological relationships in the square are showed into details in (**b**). (**b**) and (**c**) Detailed hydrogeological relationships between Calderone glacier acting as a water reservoir for the carbonate aquifer down below; i: hydraulic gradient; k: hydraulic conductivity (kh: karst horizon; lim: limestone; dol: dolomite). The hydrogeological relationships in the square are showed in detail in (**c**).

**Figure 3:**
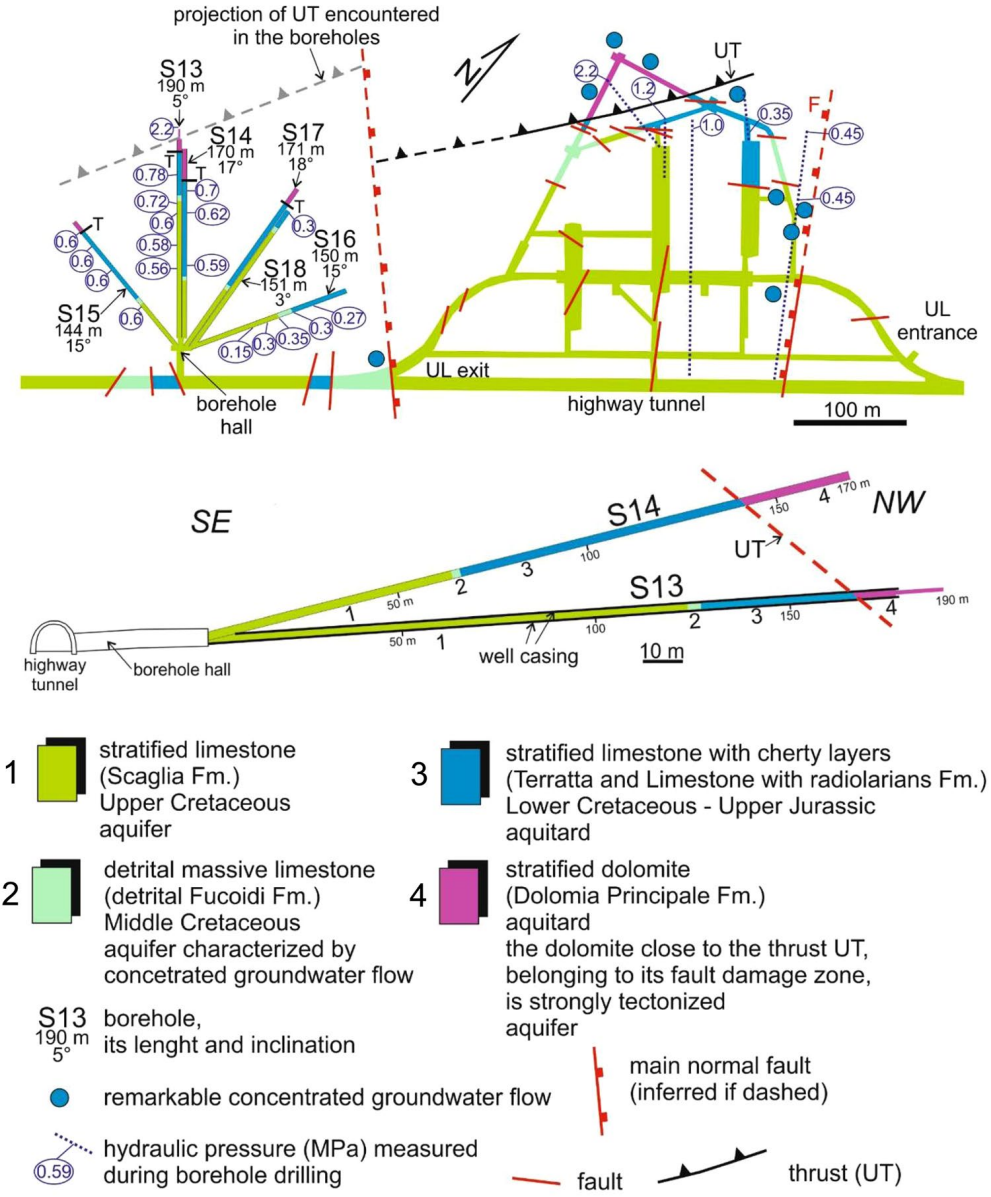
Top: geological map of underground laboratories (*LNGS-INFN*), right side, and borehole hall at left (redrawn from [54]). The figure shows the characteristic of the 6 horizontal boreholes (S13, S14, S15, S16, S17 and S18). The S13 borehole was monitored in this study. Bottom: geological logs of S13 and S14 boreholes (top scheme and Fig. 2 for location): stratified limestone (Scaglia Fm); detrital massive limestone (detrital Fucoidi Fm); stratified limestone with cherty layers (Terratta and Limestone with radiolarians Fms); stratified dolomite (Dolomia principale Fm); UT: upper thrust; UL: Underground Laboratories. The hydraulic pressure (MPa) measured during the boring are reported^54^. The black bold lines of S13 borehole represent the iron tube (150 mm of section).

We made our observations from a tunnel at 965 m a.s.l. (Fig. 2), in the core of the Gran Sasso aquifer beneath a 1,400 m-thick layer of rocks. The horizontal S13 borehole (Figs 2 and 3) was drilled around the end of the 1980s in a hall excavated in Meso-Cenozoic limestones and dolostones close to the underground laboratories of Gran Sasso (*LNGS-INFN*). It has a horizontal length of slightly over 190 m and dips gently upward by ~5° (Fig. 3). The S13 borehole intercepts a thrust fault near its end (Figs 2 and 3). The initial 175 m of S13 borehole are tubed with a well casing, while the last 10 m drain inside the upper Triassic dolomite^54,55^.

## Hydrogeological Setting

Most of the Abruzzi region is a seismic area characterized by normal faulting earthquakes reaching maximum intensity values up to *XI MCS*, corresponding to magnitudes close to 7.0; the most destructive known events occurred in 1349, 1461, 1703, 1915 and 2009^56^. The seismic activity of the Abruzzi region is quite like that of most of the central-southern Apennines, with predominant normal faulting earthquakes generated by predominant NW-SE trending fault systems of the Apenninic chain.^57–62^. The Gran Sasso chain is in the northern part of the Abruzzi region and corresponds to the main topographic and geological feature of central Apennines: its culmination (Corno Grande, 2,912 m a.s.l.) is the highest peak in peninsular Italy. The *LNGS-INFN* underground laboratories, with a rock overburden of 1,400 m, are placed in this area together with the S13 borehole site (Fig. 2). No significant historical earthquakes are known in the Gran Sasso chain, but paleoseismological studies reported multiple surface-faulting events; the magnitude of the two most recent strong events (5^th^-3^rd^ cent. BC and 6^th^-5^th^ millennium BC) have been estimated at about 7.0^63^.

During the 6 April 2009, M_w_ 6.3 L’Aquila earthquake and the related seismic sequence, several investigators studied groundwater post-seismic changes (spring discharge, water table and water content of main ions, ^222^Rn, CO_2_, water isotopes and Uranium) of the Gran Sasso aquifer^64–68^. Later, the S13 borehole was specifically selected with the goal of continuous monitoring of hydraulic pressure, electrical conductivity and temperature at a very high sampling frequency. This choice was guided by the observation that S13 borehole clearly recorded water table changes induced by the 2009 L’Aquila earthquake^64,65^.

The 1,000 km^2^-wide fractured and fault-partitioned Gran Sasso carbonate aquifer is hosting the nuclear physics underground laboratories of Gran Sasso (*LNGS-INFN*) and six horizontal perforations of the borehole hall, among which the S13 borehole (Fig. 3 for details)^54^. This aquifer is a representative carbonate aquifer of the Mediterranean domain and over the past few decades has been the subject of investigations in the framework of several projects concerning hydrogeology, hydrogeochemistry and isotope hydrology^69–78^. The Gran Sasso aquifer is composed of Meso-Cenozoic basin-to-slope and reef-platform carbonate rocks, which are arranged in a thrust belt geometry formed during the Upper Miocene Apenninc orogeny^69,70^. The thrust belt is subsequenty displaced by Plio-Quaternay normal faults generating by the NE-migration of the Tyrrhenian post-orogenic extensional front^54,55,79^. The Gran Sasso aquifer is hydraulically confined to the north and to the east by the Upper Miocene terrigenous lithologies as aquiclude. The permeability boundary is represented by the regional north-verging overthrust causing the overlaying of the Gran Sasso aquifer (in the hangingwall) onto terrigenous aquiclude (in the footwall) (Figs 2 and 3)^70,77^.

The underground laboratories (*LNGS-INFN*) and the borehole area intersect the upper thrust (UT in Figs 2 and 3). Its hangingwall and footwall are formed by lower Liassic limestones-upper Triassic dolostones and upper Cretaceous-upper Jurassic mud-supported detrital and cherty limestones, respectively^54,55^. Groundwater from the core aquifer is partially drained by the two highway tunnels and by the underground laboratories (UL LNGS in Fig. 2); it is important to stress that there are no pumping systems in the whole area, and groundwater is only passively drained from the two sides of the Gran Sasso chain^78^. Since the 1980s, groundwater flowing in the tunnels has been exploited as drinking water. The decrease of spring discharge measured in the period 1979–1990 was interpreted as a transient phenomenon caused by the tunnel excavation. Once the aquifer reached a new steady condition, however, progressive stabilization of discharge was expected^70^. Hydraulic pressure measured at the end of the 150–200 m-long horizontal boreholes falls in the range of 0.5–0.7 MPa (piezometric head of 50–70 m), except for S13 borehole, which exhibits a much higher pressure, about 2.0–2.5 MPa corresponding to a piezometric head of 200–250 m (Figs 2 and 3)^54^.

## Instrumentation

**Figure 4:**
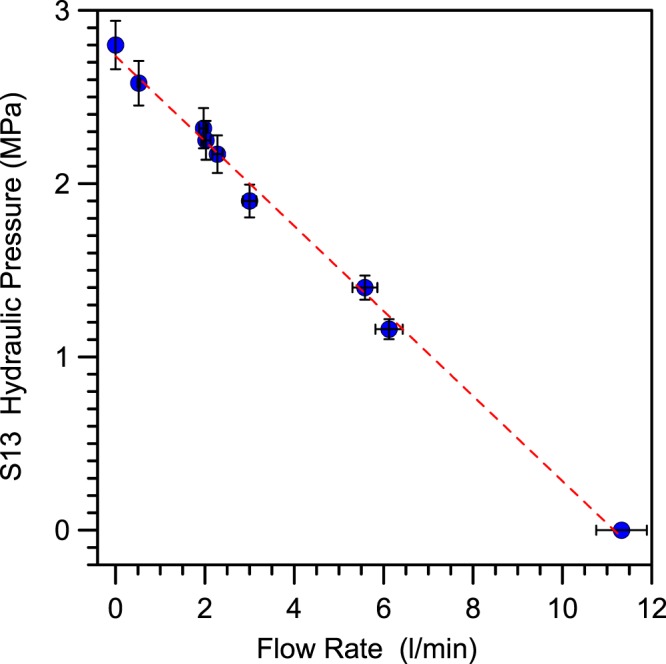
Hydraulic pressure (MPa) *vs* flow rate (l/min) of the S13 monitored borehole. The vertical and horizontal error bars are + /− 5% of value. The linear fit leads to the following equation: *P*(MPa) = −0.25*flow_rate (l/min)* + 2.7*.

**Figure 5:**
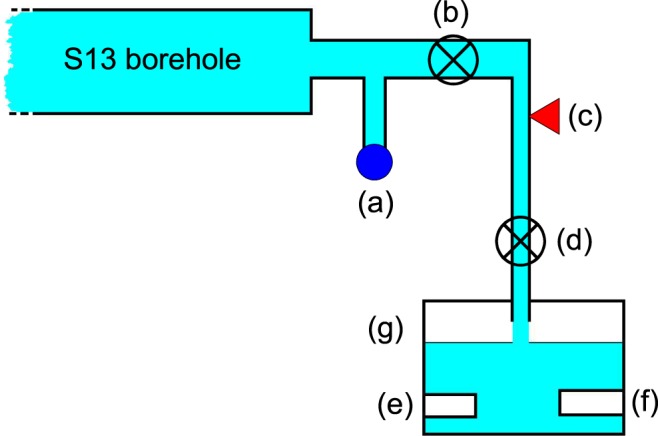
Hydraulic scheme (not in scale) of the experimental apparatus. The length of the S13 horizontal borehole is 190 m. (**a**) old analogic manometer; (**b**) hydraulic valve that is always open during the data acquisition periods; (**c**) hydraulic pressure sensor; (**d**) hydraulic valve not completely close to enable the measurement of temperature and electrical conductivity in the box (**g**); (**e**) temperature sensor; (**f**) electrical conductivity sensor; (**g**) transparent plastic container housing the temperature and electrical conductivity sensors; water is expelled when reaching about three quarters of the volume of the container.

Our experimental equipment includes a 3-channels 24-bit ADC (mod. *SL06* by Sara Electronic Instruments – http://www.sara.pg.it/) set up for continuous local recording. We started data acquisition on 1 May 2015 by continuous high-frequency sampling (10–50 Hz for each channel) of groundwater hydraulic pressure (Gems sensor, 3500 series, 0–4 MPa range), temperature (Pt1000) and electrical conductivity (www.bc-electronics.it, mod. Sl-311). From the end of 2015 the sampling rate was fixed at 20 Hz for each channel^80^. Figure 4 shows the linear behaviour and fit of hydraulic pressure *vs* its flow of S13 borehole, Fig. 5 shows a simple hydraulic scheme of the apparatus located at the S13 borehole wellhead.

The hydrological characteristics of the S13 borehole showed a specific peculiarity: high hydraulic pressure with respect to very low flow rate (Fig. 4). To measure temperature and electrical conductivity we needed to spill some water, but to avoid disturbance in hydraulic pressure measurement we decided to limit as much as possible the spilled flow. To this end, we installed the hydraulic system shown in Fig. 5; a manual hydraulic valve (d) in Fig. 5 permitted to regulate the water flow. The hydraulic pressure sensor was connected directly to the S13 horizontal borehole output, (c) in Fig. 5, while the other two sensors (temperature (e) and electrical conductivity (f) devices in Fig. 5) are immersed in a small box fed, (g) in Fig. 5, by a continuous supply of water at atmospheric pressure^80^.

**Figure 6:**
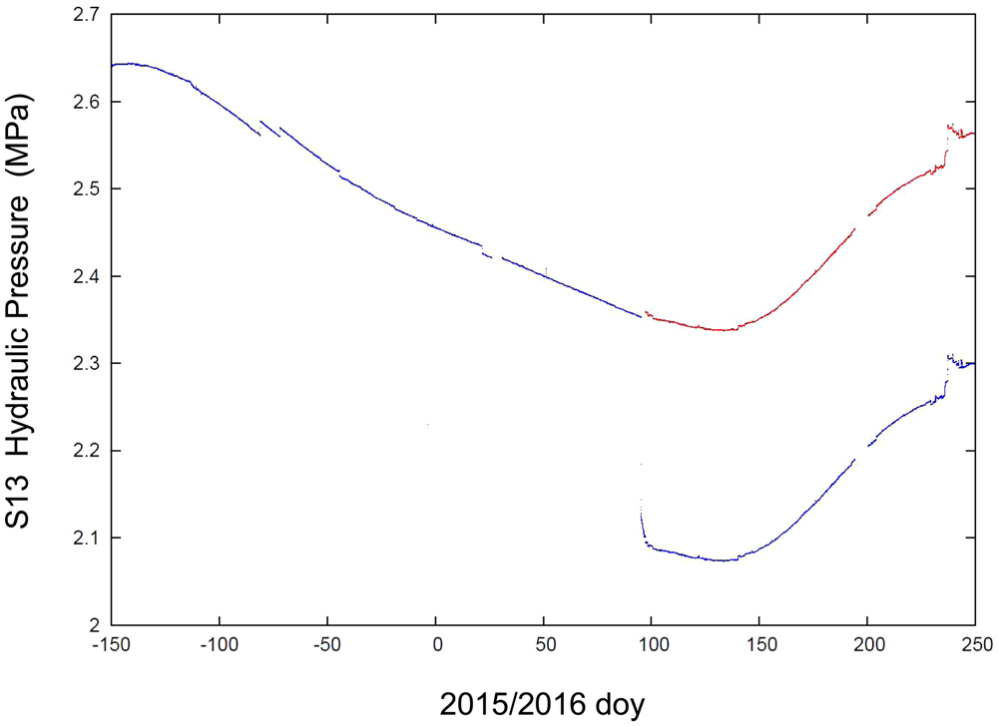
Hydraulic pressure (MPa) plots. Data from 03 August 2015 (doy 215) to 5 September 2016 (doy 249), about 400 days of data. At the begin of April 2016, we increased flow rate and the hydraulic pressure decreased from 2.35 MPa to 2.08 MPa. The red plot is only the translation of the bottom black line.

We decided to work with a flow rate of 0.85 l/min (from May 2015) and then we increased it on 5 April 2016 to 1.97 l/min. The increased flow rate carried out on 5 April 2016 resulted in a pressure drop of about 0.3 MPa (Fig. 6). Under these conditions of high pressures and outlet of water from an extremely small opening, the flow rate was not in a laminar regime but in a fully developed turbulence.

A seismic station of the national network^53^ of Istituto Nazionale di Geofisica e Vulcanologia (*INGV*) is located about 250 m from the S13 borehole inside the underground laboratory of Gran Sasso (*LNGS-INFN*). The seismic station (international code: *GIGS*) was deployed in the framework of the *GINGER* experiment^81–83^ and is equipped with two broadband seismometers, including a Nanometrics Trillium 240 s (see http://iside.rm.ingv.it/iside/standard/info_stazione.jsp?page=sta&sta=2571 for further details) and a Guralp CMG 3 T 360 s. This instrumentation is used both for continuous microseismic monitoring of the Gran Sasso chain and for recording global seismicity.

## Data Discussion and Interpretation

**Figure 7:**
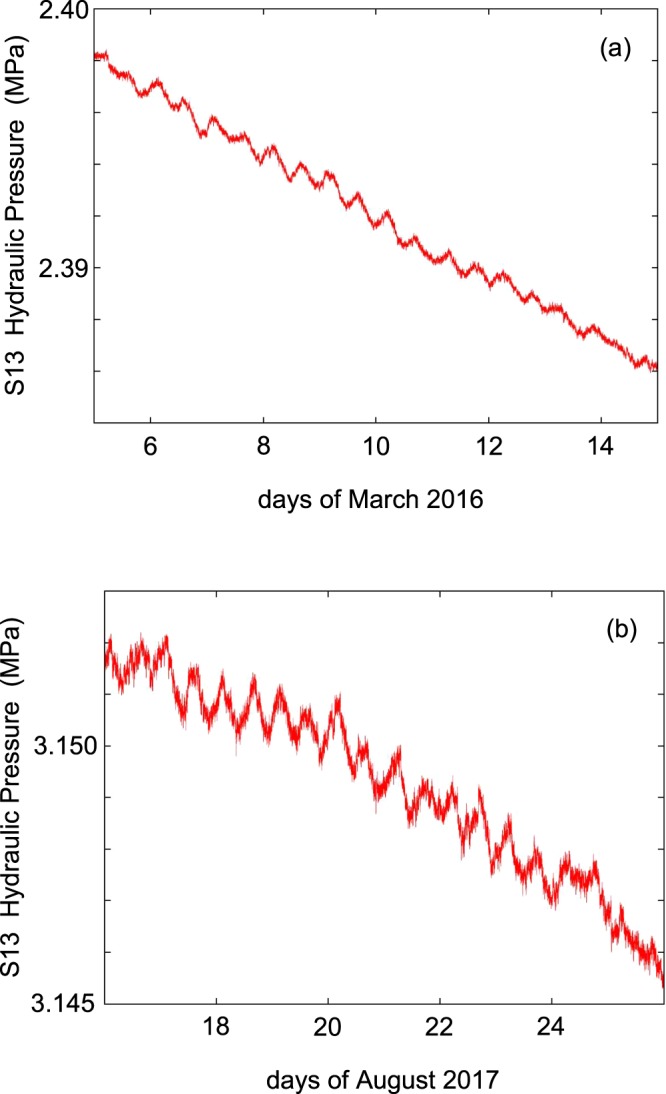
Tidal signal in the hydraulic pressure data of S13 borehole. (**a**) data from 5 to 15 March 2016, about 6 months before the Amatrice earthquake; (**b**) data from 16 to 26 August 2017, about one year after the Amatrice earthquake.

During the months of continuous monitoring (May 2015–Jan 2017) the hydraulic pressure signal of the S13 horizontal borehole showed the recharge and depletion cycle of the Gran Sasso aquifer (Fig. 6) and displayed also the sun/moon tides (Fig. 7). The temperature followed both seasonal and daily variations, the former linked always to the hydrogeological cycle, the latter due to the closeness of the highway tunnel with the S13 hall, while the electrical conductivity did not show any significant periodical variations. Apart from these expected trends, at the end of 2015 the hydraulic pressure data showed coseismic effects related to two large earthquakes occurring during the monitored period, respectively located at global and regional distance: the 16 Sept 2015, Illapel, central Chile earthquake (M_w_ 8.3, distance about of 12,000 km), and the 17 Nov 2015, Lefkada, western Greece earthquake (M_w_ 6.5, distance about 700 km). Details of the waveforms recorded can be found in [80]; the arrival of Love and Rayleigh waves from the Chile earthquake and P- and S- waves from the Greece earthquake are very clear^80^. The signal recorded in the pressure channel during the earthquake represents the response induced by wave arrivals exciting the whole aquifer.

A few days after the 24 August 2016 (01:36:32 UT, doy 237) Amatrice earthquake (M_w_ 6.0), we recovered our data to search for any changes possibly induced by the earthquake. We expected the signals to be large due the relative closeness of the Amatrice event (about 39 km to the monitored site) and its notable magnitude. As we observed an unexpected behaviour only in the hydraulic pressure and electrical conductivity channels, in the following we will refer only to these observations.

**Figure 8:**
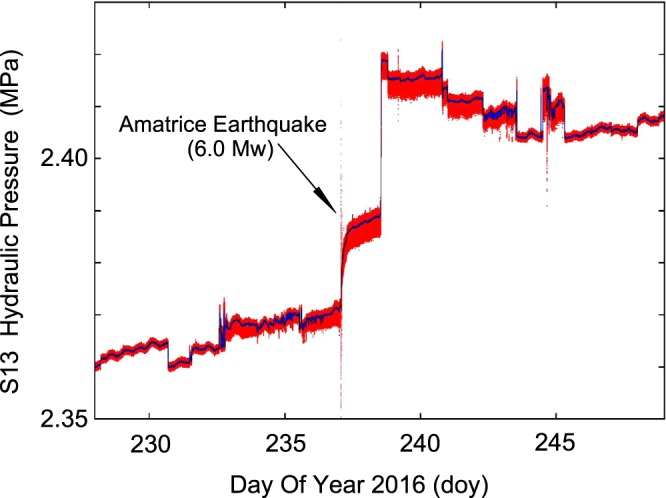
Plot of the hydraulic pressure (in MPa) of S13 borehole, red line, at 20 Hz sampling rate (from 15 August, doy 228, to 4 September, doy 248, 2016). The blue line represents the 60 s moving average.

**Figure 9:**
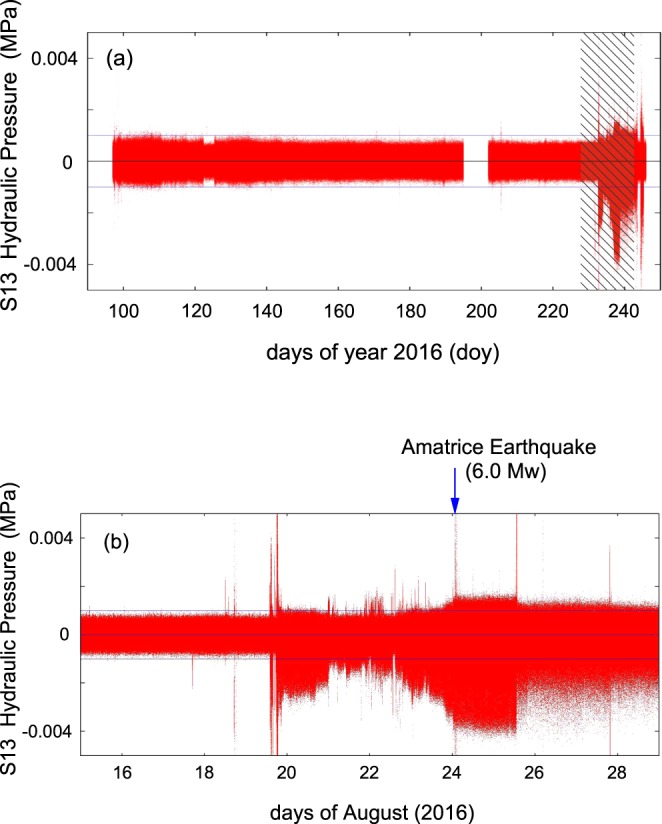
(**a**) Detrended of hydraulic pressure of S13 borehole from April to August 2016. (**b**) Detrended of hydraulic pressure of S13 borehole from 15 (doy 228) to 29 August 2016 (doy 242), shaded rectangle in (a).The blue arrow in the top of (b) represents the Amatrice earthquake occurrence. In the period from May 2015 to March 2016 we did not observe any variations.

**Figure 10:**
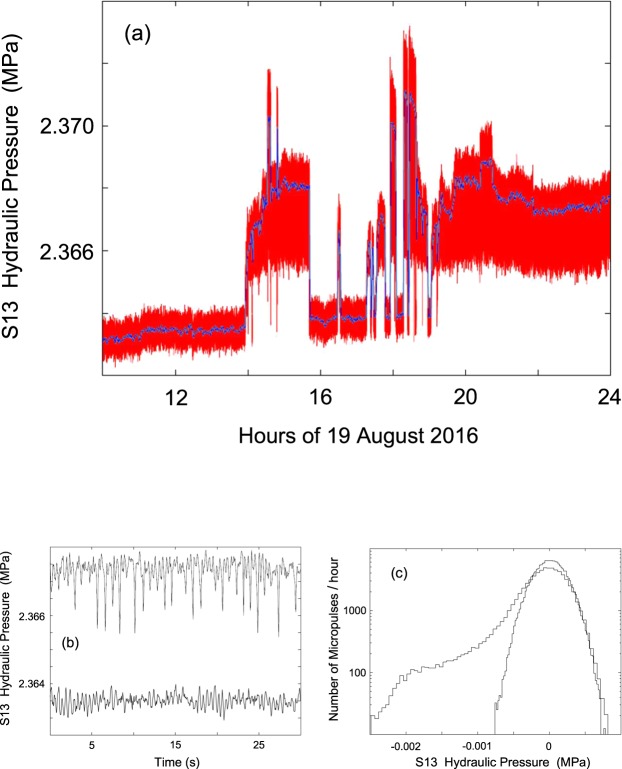
(**a**) Plot of the hydraulic pressure (in MPa) at 20 Hz sampling rate recorded on 19 August 2016 (doy 232) from 10:00 to 24:00 UT (red line). The blue line represents the 60 s moving average. (**b**) A 30 s section of recorded data: after 13:00 UT (lower line) and after 22:00 UT (upper line) of 19 August 2016. (**c**) Histograms of the data distributions: the asymmetric distribution refers to one hour of recording from 15:00 to 16:00 UT of 20 August 2016 (doy 233), while the symmetric distribution refers to one hour from 12:00 to 13:00 UT of 19 August 2016 (doy 232).

**Figure 11:**
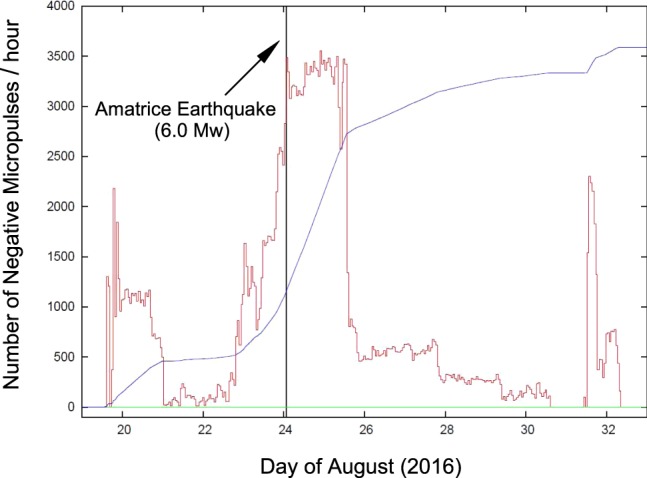
Number of negative micropulses (red line) each hour from 19 August (doy 232) to 2 September 2016 (doy 246), while the blue line shows the integral trend in arbitrary scale, black vertical line represents the Amatrice earthquake occurrence and green horizontal line is the zero level. In the period from May 2015 to July 2016 we did not observe any negative micropulses.

Figure 8 shows unprocessed data (at a 20 Hz sampling rate, red line) of the hydraulic pressure from 15 August (doy 229) to 5 September 2016 (doy 249) using a 60 s moving average (blue line). The presence of the average values reveals the structural change of the signal (asymmetry) that will be further illustrated in Figs 9–11 and 13. In the first days (15 August–18 August 2016) we observed the aquifer recharge superimposed on a small tidal modulation; a similar behaviour was seen again only around the end of August. Between 19 August – i.e. a week before the earthquake – and the end of August 2016 we observed a different regime of amplitude fluctuations of the hydraulic pressure. To emphasize this effect, we performed a detrend analysis in hydraulic pressure data for the May 2015 – Sept 2016 period. Figure 9a shows the detrended of hydraulic pressure of S13 borehole from April to August 2016 (about 150 days) while the Fig. 9b shows the detrend analysis only from 15 August (doy 228) to 29 August 2016 (doy 242). From the start of data collection (May 2015), detrended data fell inside the +/−0.001 MPa band (signalled by blue lines in Fig. 9a,b). This was true until 19 August 2016 (five days before Amatrice earthquake) when large and asymmetric fluctuations appeared, lasting till the end of August 2016 (Figs 8 and 9b). In the period from May 2015 to July 2016 we did not observe any variations. In Fig. 8 and in a minor way also in Fig. 6, moreover, we observe a variety of steps. In most cases the steps are up or down with durations of less than 1 minute. These are probably related to pore-pressure phenomena. Coseismic steps are comparatively slower (about ten minutes)^9,13,16,18–21,23–25^.

We next focus on the regime change that occurred on 19 August 2016 (doy 232) and on the resulting anomalies in the hydraulic pressure signal characteristics from S13 borehole. Figure 10a shows the onset of this anomalous behaviour on 19 August 2016. A transition from one regime to a different regime was recorded over a period of about 6 hours (from 14:00 to 20:00 UT). This change occurred not progressively but following a series of *“flip-flop”* cycles. This change is marked by two distinct features: an increase in the average hydraulic pressure (about 0.004 MPa), and a more dramatic change in the fine structure of the signal. The latter effect can be better appreciated by looking at the two panels (Fig. 10b,c) representing the behaviour of hydraulic pressure data before and after the transition. The presence of asymmetric fluctuations (negative micropulses) is evident following the transition (Fig. 10b). This behaviour is shown more quantitatively in the histograms displayed in Fig. 10c. From 19 August 2016 (doy 232) onwards, this behaviour is observed systematically, although to a varying degree. Figure 11 shows the hourly number of negative micropulses (red line) from 19 August to 2 September 2016, while the blue line shows the integral trend on an arbitrary scale. It is of interest to note that, starting from about 36 hours before the 24 August mainshock there is a large increase of negative micropulses, culminating at the time of occurrence of the Amatrice earthquake. Moreover, in the period from May 2015 to July 2016 we did not observe any negative micropulses.

To explain the presence of these negative micropulses we go back to the details of the hydraulic setup. The flux of water from the S13 borehole is determined by the balance of two effects: the high impedance of the source associated with the high level of fracturing of the rocks crossed by the S13 borehole, and the small area of the valve orifice (O(1) mm^2^). The density and viscosity of the water limit the flow rate to about 2 l/min with an inlet/outlet pressure ratio of about 25 (and flow speed of about 10 m/s). Any increase in flow rate implies a sudden decrease of hydraulic pressure as showed by the trend of Fig. 4. Then when a macroscopic bubble (O(1) ml at 1 bar) of geogas reaches the orifice of the valve an instantaneous increase of flow rate occurs due to the sudden decrease of density and viscosity. This results in a rapid decrease of the hydraulic pressure, as measured above the valve, producing a negative micropulse in our recording (on the order of 2 × 10^−3^ MPa). Therefore, the counting rate of negative micropulses is a proxy for the abundance of gas bubbles in groundwater.

**Figure 12:**
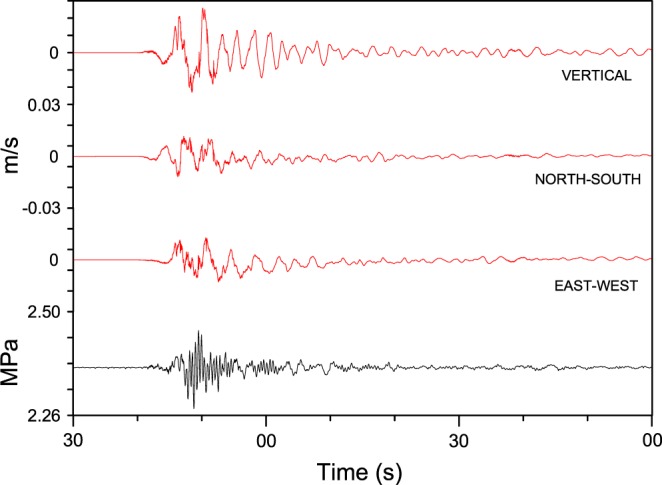
Waveforms corresponding to the Amatrice earthquake, 24 August 2016, 01:36:32 UT, doy 237, (M_w_ 6.0); the red lines represent the vertical, north-south and east-west components of broadband seismic station (GIGS) equipped with a Trillium 240 s seismometer; the black line is the hydraulic pressure expressed in MPa. To note the remarkable variation (0.2 Mpa pp) during the S-waves arrival. The time scale begins at 01:36:30 (UT) on 24 August 2016.

Figure 12 focuses on 24 August 2016 from 01:36:30 to 01:38:00 UT, when the Amatrice earthquake occurred. Data from the 3-components *GIGS* broadband seismic station are shown (red lines) next to the hydraulic pressure signal (black line) from S13 borehole as a reference. Figure 12 shows a main feature: the remarkable variation of hydraulic pressure (0.2 MPa pp) following the arrival of the S-waves; afterwards, a slow increase of about 0.02 MPa (corresponding to a piezometric head change of about 2 m) were recorded over a period of about 5 hours following the end of the earthquake signal (Fig. 8). Although similar observations have already been described in the literature^9,84^, the results we obtained exhibit a remarkable magnitude and a great level of detail^80^. The novelty of our time series of hydraulic pressure lies also in the presence of unambiguous signals starting several days before the mainshock of 24 August 2016.

**Figure 13:**
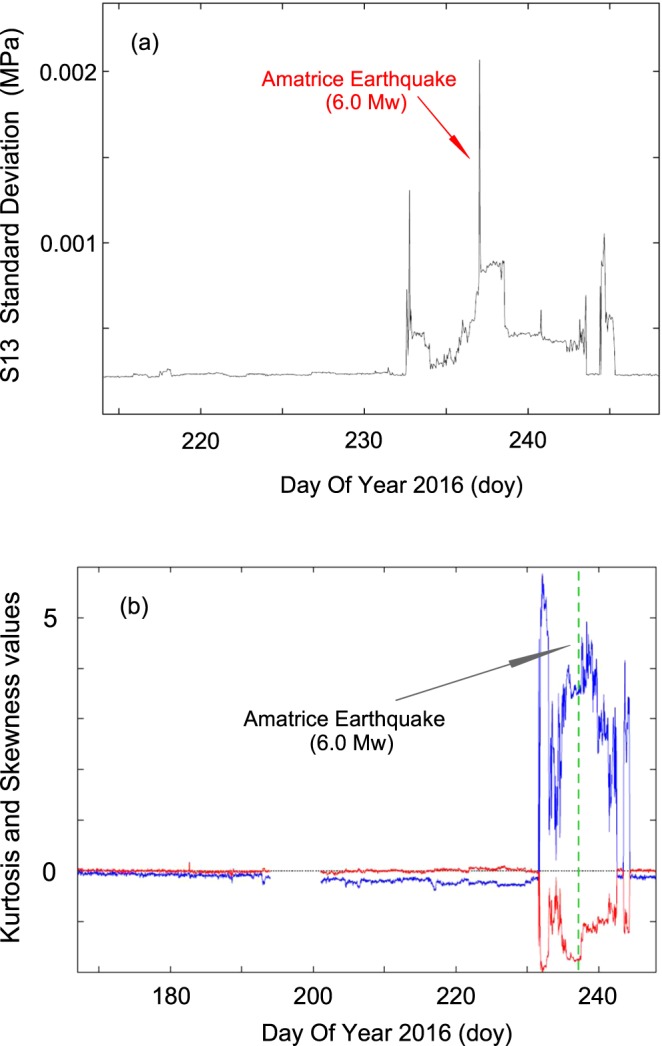
Statistical analysis: (**a**) standard deviation from 27 July (doy 209) to 4 September 2016 (doy 248) from hydraulic pressure data of S13 borehole; (**b**) kurtosis (blue) and skewness (red) values from 15 June (doy 167) to 4 September 2016 (doy 248) from hydraulic pressure data of S13 borehole. The statistical indicators are evaluated each 60 s. The plotted values are one-hour averages. Data missing (12–19 July 2016, doy 194–201) is due to instrumentation maintenance. The Amatrice earthquake (M_w_ 6.0) occurs on 24 August 2016 (doy 237) at 01:36:32 UT.

To investigate these rather unexpected phenomena in a quantitative fashion, we performed an elementary statistical analysis of the hydraulic pressure time series looking at the first three moments of the complete time-series. More specifically, after subtracting the (60 s) moving average we evaluated the standard deviation (*m*_2_)^*1/2*^, the skewness *m*_3_*/(m*_2_)^*3/2*^ and the kurtosis (*m*_4_*/m*_2_^2^ *– 3*) of the resulting distribution. Figures 13a,b show the results of the statistical analysis of hydraulic pressure data from S13 borehole. The standard deviation (Fig. 13a) is computed from 27 July (doy 209) to 4 September 2016 (doy 248). The Kurtosis and Skewness (Fig. 13b) values are computed from 15 June (doy 167) to 4 September 2016 (doy 248). The evidence of a signal change was very clear about five days before the main shock, although the change was somewhat detectable starting 40 days before the earthquake when a slow decrease in kurtosis is particularly evident. We checked the relevance of this signal by looking at the kurtosis of the full dataset (1 May 2015 to 5 September 2016) and we found no significant deviation from the zero value until 40 days before the main shock. It is worth to note that the 24 August mainshock was not preceded by any sizable foreshock; particularly, in the circular area of radius 10 km from the M_w_ 6.0 epicenter we counted only one event with M_w_ greater to 1.8 (15 Aug 2016, doy 228, at 19:00:41 UT – M_w_ 1.9) in the period 1 May – 24 August 2016^53^.

**Figure 14:**
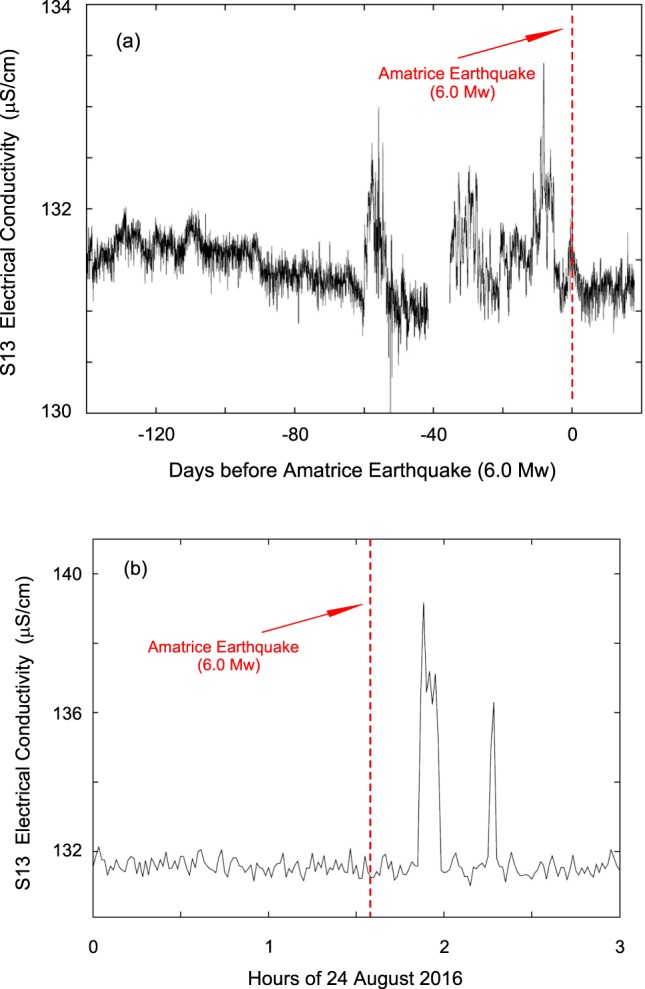
Electrical conductivity data of S13 borehole from 140 days before the Amatrice earthquake (24 August 2016 (doy 237) at 01:36:32 UT). The anomaly starts about 60 days before the mainshock in accordance with weak deviation from zero of Kurtosis of hydraulic pressure data in Fig. 13 (blue line); in the period from May 2015 to May 2016 we did not observe any significant variation. (**b**) Electrical conductivity data of Amatrice earthquake of 24 August 2016 (01:36:32 UT) from 00:00 to 03:00 UT.

We next address the electrical conductivity data. Figure 14a shows the electrical conductivity signal averaged over 1 hour, starting 140 days before the Amatrice earthquake (occurring at the 0 in Fig. 14a) and lasting 20 days after. A significant change was noted starting 60 days before the mainshock, which can be correlated with the kurtosis analysis of hydraulic pressure (blue line in Fig. 13b). In the period from May 2015 to May 2016 we did not observe any significant variation of electrical conductivity; note again that no significant foreshocks or swarms were present in the area. Figure 14b shows a zoom of three hours (from 00:00 to 03:00 UT of 24 August 2016) of electrical conductivity data averaged every minute; a change in conductivity occurred at 01:51 UT, and the Amatrice earthquake strikes at 01:36. There were 15 minutes of lag time, but considering that the horizontal S13 tube contains about 2,000 l of water flowing at 2 l per minute, this was traced back, to the upper thrust zone, to around 17 hours before the mainshock.

**Figure 15:**
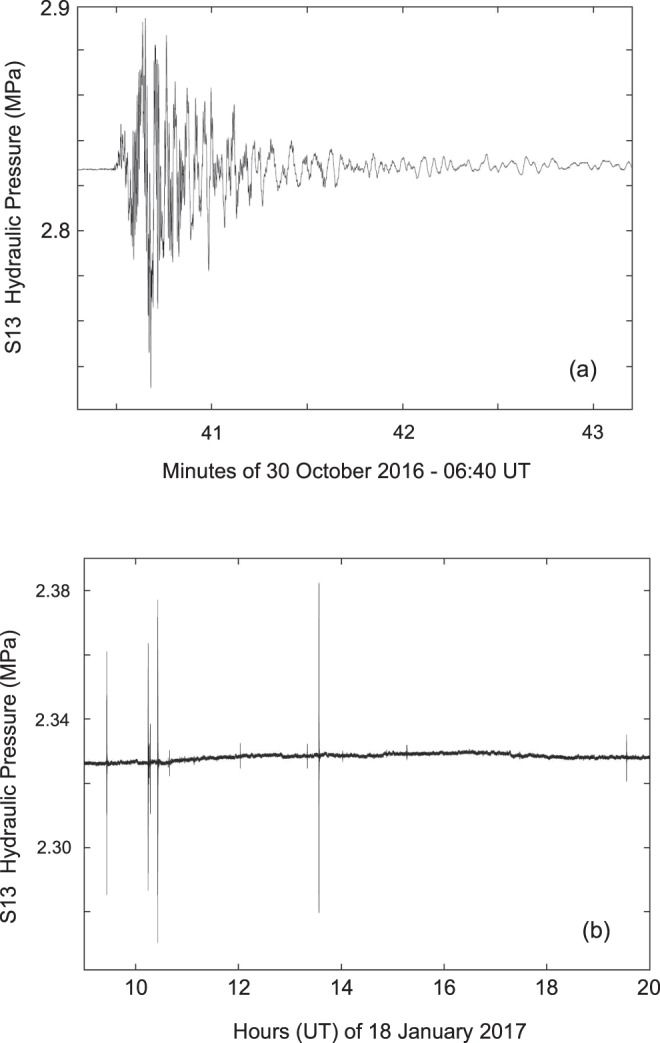
(**a**) Plot of the hydraulic pressure (in MPa) of S13 borehole at 20 Hz of sampling rate for 30 October 2016 earthquake, M_w_ 6.5, at 06:40:17 UT. (**b**) Plot of the hydraulic pressure (in MPa) of S13 borehole at 20 Hz of sampling rate for the sequence of 18 January 2017 (data from 08:00 to 20:00 UT); the main events are: 09:25:40 UT (M_w_ 5.1), 10:14:10 UT (M_w_ 5.5), 10:25:24 UT (M_w_ 5.4) and 13:33:37 UT (M_w_ 5.0)^53^. It is also possible to note a small upward coseismic offset of about 0.002 MPa, as well as a dozen earthquakes of minor magnitude.

During the period of data analysis and interpretation (between September and the middle of October 2016), which was taken a few months, we made further modifications and tests of our hydraulic system. At the end of October, the seismic sequence restarted with two events of magnitude greater than 5.0 (26 October 2016: 5.4 M_w_ at 17:10:36 UT and 5.9 M_w_ at 19:18:07 UT) culminating on October 30, 2016 with a 6.5 M_w_ (06:40:17 UT)^53^. During this period, only the pressure sensor was continuously recording, but with a different hydraulic port setup, so a direct comparison with older data is difficult. Figure 15a shows the 30 October earthquake (6.5 M_w_ at 06:40:17 UT) and Fig. 15b the sequence of 18 January 2017^53^ (data from 06:00 to 18:00 UT); the main events are: 09:25:40 UT (M_w_ 5.1), 10:14:10 UT (M_w_ 5.5), 10:25:24 UT (M_w_ 5.4) and 13:33:37 UT (M_w_ 5.0). The amplitudes of four events with M_w_ > 5 are not directly correlated with magnitude because the distance between the earthquake epicentre and the S13 site decreases (about 26.6, 25.5, 25.1 and 24.6 km respectively); in addition, it is easy to notice the presence of a dozen earthquakes of smaller magnitude.

**Figure 16:**
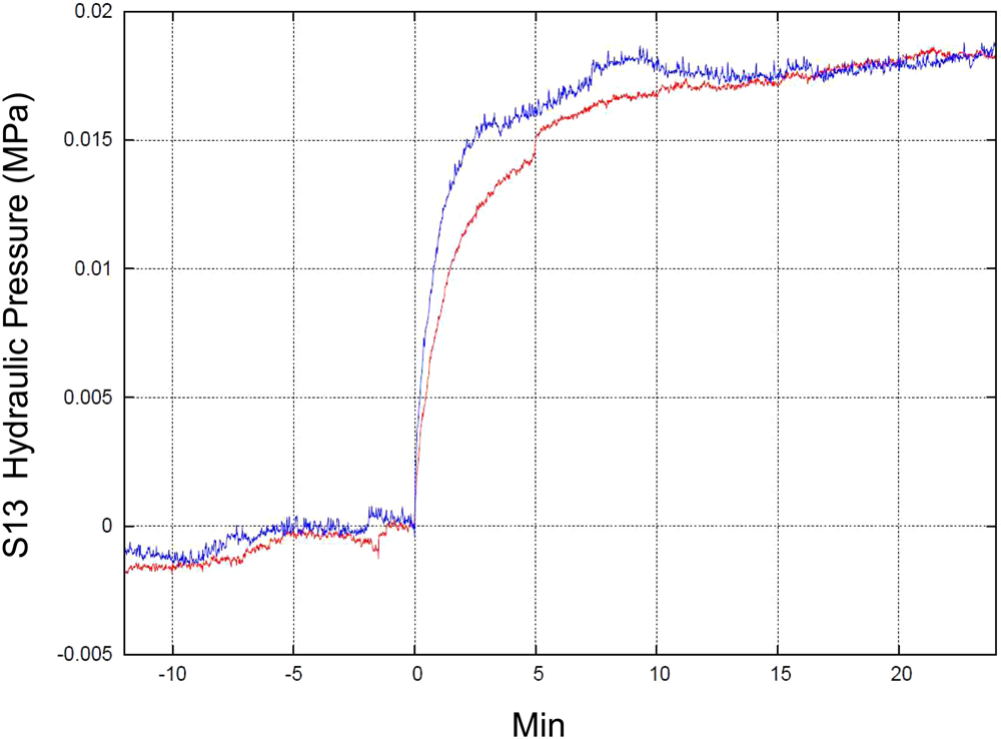
Superimposed plots of the hydraulic pressure of S13 borehole (in MPa), with moving average at 1 minute, of 24 August 2016, M_w_ 6.0 (red line) and 30 October 2016, M_w_ 6.5 (blue line) earthquakes (the average smooths the elastic part of earthquake signals). The upward coseismic offset is the same (data are shifted only in time and hydraulic pressure level).

The only realistic comparison that can be made between these two periods concerns the coseismic effect following the two mainshocks of 24 August (6.0 M_w_) and 30 October 2016 (6.5 M_w_). The upward coseismic offset related to the two earthquakes is clearly shown by the superimposed plots of the hydraulic pressure (in MPa), with moving average at 1 minute (the average smoothes out the elastic part of earthquake signals, principally P-, S- and Coda- waves) (Fig. 16). The positive coseismic offset is almost the same (the data are aligned and superimposed forcing at (0,0) the start of two earthquakes) (Fig. 16).

## Conclusions

The presence of the S13 horizontal borehole near the underground laboratories of Gran Sasso (*LNGS-INFN*) in central Italy, gave us the unique opportunity to investigate the pristine groundwater reservoir in the core of the Gran Sasso aquifer located in the very seismically active area of central Apennines (Fig. 1). The S13 horizontal borehole was drilled at the end of the 1980s during excavation works for a tunnel across the Gran Sasso chain, located about 39 km south-eastward from the Amatrice earthquake epicenter (M_w_ 6.0) (Fig. 3).

Starting in May 2015, we used the S13 borehole to measure continuously hydraulic pressure, groundwater temperature and electrical conductivity with a high sampling rate (10 Hz to 50 Hz) for each channel. Monitoring was still ongoing when the 24 August 2016 (01:36:32 UT, doy 237), Amatrice earthquake (M_w_ 6.0) hit about 39 km from the study site (Figs 1–3). The acquired record showed distinct and interesting signals in the hydraulic pressure and electrical conductivity data of the Gran Sasso groundwater before, during and after this earthquake. We recognized anomalies that can be classified both as long-term seismic precursors (anomalies of hydraulic pressure signals and electrical conductivity about 40–60 days before the Amatrice earthquake – Figs 13 and 14) and as short-term seismic precursors (about 5 days in the hydraulic pressure data – Figs 8, 9, 11 and 13).

For many years, scientists have observed changes in the amount, direction, and discharge of water flow in streams and in the water level of wells. Furthermore, changes in fluid pressure in the subsurface have been documented following large earthquakes by observing fluctuations as large as several meters in water levels resulting from mid-size regional earthquakes or even from very large earthquakes located thousands of kilometers away. The authors of ref.^9^ for example, have supplied a review of water level variations in surface wells coincident with the arrival of teleseismic waves. Our data, however, represent the first observations of earthquake-related signals in hydraulic pressure measured with continuous high-frequency sample rate (20 Hz) monitoring of the S13 borehole placed deep within a Gran Sasso carbonate aquifer; S13 also intersects a thrust fault near its end (Figs 2 and 3).

Groundwater variations are believed to respond to crustal deformation processes well before the occurrence of significant earthquakes^20,21^. Several mechanisms have been proposed to explain such variations during the seismic cycle, including the dilatancy theory which assumes that, during the pre-seismic phase, the rock volume increase, prior to rupture, is favoured by the micro-fracturing within the earthquake source volume. Micro-fracturing induces also rock permeability increase favouring groundwater variations and mobilization or uprising of geogas^9,50,85^.

Groundwater monitoring at the S13 borehole revealed the existence of highly dynamic aquifer behaviour. The data collected during these twenty-one months turned out to be quite a useful tool to: (*i*) characterize the background in groundwater monitoring, and (*ii*) identify more clearly the observed anomalies and their possible link with phenomena of tectonic interest. In this respect, the anomalies observed in the hydraulic pressure 5 days before the 24 August 2016, Amatrice earthquake are obvious.

We interpret the presence of negative micropulses in hydraulic pressure data (Fig. 10b) as the result of a possible uprising of deep geogas, mainly CO_2_, consistent with recent studies suggesting a link between fault mechanics, seismicity, underground fluids dynamics and CO_2_ uprising^86–94^. More precisely, during the L’Aquila earthquake (April 6^th^, 2009, 6.3 M_w_), the evolution of seismicity was being driven in part by the poro-elastic response of trapped reservoirs of high-pressure fluid, presumably CO_2_, and postseismic fluid flow initiated by the main shock^44^.

Further investigation of the relationship between earthquakes and changes in groundwater parameters near large seismogenic faults, are needed for a full understanding of preseismic, coseismic and postseismic processes.

## References

[1] Cicerone RD, Ebel JE, Britton J (2009). A systematic compilation of earthquake precursors. Tectonophysics.

[2] Wyss M (1997). Cannot earthquakes be predicted?. Science.

[3] Scholz CH, Sykes LR, Aggarwal YP (1973). Earthquake Prediction: A Physical Basis. Science.

[4] Wyss M, Booth DC (1997). The IASPEI procedure for the evaluation of earthquake precursors. Geophys. J. Int..

[5] Geller RJ (1997). Earthquake prediction: a critical review. Geophys. J. Int..

[6] Kagan YY (1997). Are earthquakes predictable?. Geophys. J. Int..

[7] Nature debates, http://www.nature.com/nature/debates/earthquake/index.html.

[8] Geller RJ, Jackson DD, Kagan YY, Mulargia F (1997). Earthquakes cannot be predicted. Science.

[9] Manga M., Wang C.-Y. (2015). Earthquake Hydrology. Treatise on Geophysics.

[10] Roeloffs EA (1988). Hydrologic precursors to earthquakes: A review. Pure and Applied Geophysics.

[11] Kissin IG, Grinevsky AO (1990). Main features of hydrogeodynamic earthquake precursors. Tectonophysics.

[12] Tsunogai U, Wakita H (1995). Precursory Chemical Changes in Ground Water: Kobe Earthquake, Japan. Science.

[13] Shi Z, Wang G, Manga M, Wang C-Y (2015). Continental-scale water-level response to a large earthquake. Geofluids.

[14] Ingebritsen SE, Manga M (2014). Earthquakes: Hydrogeochemical precursors. Nature Geoscience.

[15] Claesson L (2004). Hydrogeochemical changes before and after a major earthquake. Geology.

[16] Chen C-H (2013). Anomalous frequency characteristics of groundwater level before major earthquakes in Taiwan. Hydrology and Earth System Sciences.

[17] Skelton A (2014). Changes in groundwater chemistry before two consecutive earthquakes in Iceland. Nature Geoscience.

[18] Roeloffs E, Quilty E (1997). Case 21: Water level and strain changes preceding and following the August 4, 1985 Kettleman Hills, California, earthquake. Pure and Applied Geophysics.

[19] Roeloffs EA (1998). Persistent water level changes in a well near Parkfield, California, due to local and distant earthquakes. J. Geophys. Res..

[20] King CY (2000). In search of earthquake precursors in the water-level data of 16 closely clustered wells at Tono, Japan. Geophys. J. Int..

[21] King CY (1999). Earthquake related water-level changes at 16 closely clustered wells in Tono, Central Japan. J. Geophys. Res..

[22] King CY, Koizumi N, Kitagawa Y (1995). Hydrogeochemical anomalies and the 1995 Kobe earthquake. Science.

[23] Chia Y (2008). Implications of coseismic groundwater level changes observed at multiple-well monitoring stations. Geophys. J. Int..

[24] Chia Y, Chiu JJ, Chiang Y-H, Lee T-P, Liu C-W (2008). Spatial and temporal changes of groundwater level induced by thrust faulting. Pure and Applied Geophysics.

[25] Montgomery DR, Manga M (2003). Stream flow and water well responses to earthquakes. Science.

[26] Muir-Wood R, King GCP (1993). Hydrological Signatures of Earthquake Strain. J. Geophys. Res..

[27] Brodsky EE, Roeloffs E, Woodcock D, Gall I, Manga M (2003). A mechanism for sustained groundwater pressure changes induced by distant earthquakes. J. Geophys. Res..

[28] Wakita H (1975). Water wells as possible indicators of tectonic strain. Science.

[29] Doglioni C, Barba S, Carminati E, Riguzzi F (2014). Fault on-off versus coseismic fluids reaction. Geoscience Frontiers.

[30] Chiarabba C, Chiodini G (2013). Continental delamination and mantle dynamics drive topography, extension and fluid discharge in the Apennines. Geology.

[31] McCaig A (1989). Fluid flow through fault zones. Nature.

[32] Barnhoorn A, Cox SF, Robinson DJ, Senden T (2010). Stress- and fluid-driven failure during fracture array growth: Implications for coupled deformation and fluid flow in the crust. Geology.

[33] Di Toro G (2011). Fault lubrication during earthquakes. Nature.

[34] Violay M (2013). Effect of water on the frictional behaviour of cohesive rocks during earthquakes. Geology.

[35] Hirth G, Beeler NM (2015). The role of fluid pressure on frictional behaviour at the base of the seismogenic zone. Geology.

[36] Sibson RH (2000). Fluid involvement in normal faulting. J. Geodynamics.

[37] Scuderia MM, Collettinia C, Marone C (2017). Frictional stability and earthquake triggering during fluid pressure stimulation of an experimental fault. Earth Planetary Science Letters.

[38] Chiarabba C (2009). The2009 L’Aquila (central Italy) Mw 6.3 earthquake: Main shock and aftershocks. Geophys. Res. Lett..

[39] Valoroso L (2010). Radiography of a normal fault system by 64,000 high-precision earthquake locations: The 2009 L’Aquila (central Italy) case study. J. Geophys. Res..

[40] Anzidei, M. & Pondrelli, S. (eds). The Amatrice seismic sequence: preliminary data and results. *Annals of Geophysics, Special Issue* 59, Fast Track5, http://www.annalsofgeophysics.eu/index.php/annals/issue/view/515 (2016).

[41] Chiaraluce L (2017). The2016 Central Italy Seismic Sequence: A First Look at the Mainshocks, Aftershocks, and Source Models. Seismological Research Letters.

[42] De Luca G (2011). La rete sismica regionale Abruzzo e sua integrazione con la RSN. Riassunti estesi del I° Workshop Tecnico Monitoraggio sismico del territorio nazionale: stato dell’arte e sviluppo delle reti di monitoraggio sismico. AA. VV. a cura di Cattaneo M. & Moretti M. – Miscellanea INGV. Roma 20-21 dicembre.

[43] Delladio A (2011). Monitoraggio sismico del territorio nazionale. Riassunti estesi del I° Workshop Tecnico Monitoraggio sismico del territorio nazionale: stato dell’arte e sviluppo delle reti di monitoraggio sismico. AA. VV. a cura di Cattaneo M. & Moretti M. – Miscellanea INGV. Roma 20-21 dicembre.

[44] Terakawa T, Zoporowski A, Galvan B, Miller SA (2010). High-pressure fluid at hypocentral depths in the L’Aquila region inferred from earthquake focal mechanisms. Geology.

[45] Savage MK (2010). The role of fluids in earthquake generation in the 2009 M_w_ 6.3 L’Aquila, Italy, earthquake and its foreshocks. Geology.

[46] Di Luccio F, Ventura G, Di Giovambattista R, Piscini A, Cinti FR (2010). Normal faults and thrusts reactivated by deep fluids: The 6 April 2009 M_w_ 6.3 L’Aquila earthquake, central Italy. J. Geophys. Res..

[47] Malagnini L, Lucente FP, De Gori P, Akinci A, Munafo I (2012). Control of pore fluid pressure diffusion on fault failure mode: Insights from the 2009 L’Aquila seismic sequence. J. Geophys. Res..

[48] Lucente FP (2010). Temporal variation of seismic velocity and anisotropy before the 2009 M_w_ 6.3 L’Aquila earthquake, Italy. Geology.

[49] Borghi A, Aoudia A, Javed F, Barzaghi R (2016). Precursory slow-slip loaded the 2009 L’Aquila earthquake sequence. Geophys. J. Int..

[50] Moro M (2017). New insights into earthquake precursors from InSAR. Scientific Reports.

[51] Barberio MD, Barbieri M, Billi A, Doglioni C, Petitta M (2017). Hydrogeochemical changes before and during the 2016 Amatrice-Norcia seismic sequence (central Italy). Scientific Reports.

[52] Petitta M (2017). Water table and discharge changes associated with the 2016-2017 seismic sequence in central Italy: hydrogeological data and conceptual model for fractured carbonate aquifers. Hydrogeology Journal.

[53] ISIDe working group – version 1.0, http://cnt.rm.ingv.it/iside, 10.13127/ISIDe (2016).

[54] Catalano, P. G. & Salza, R. Laboratori sotterranei. Relazione idrogeologica e sistemi di drenaggio e canalizzazione. [INFN underground laboratories. Hydrogeology and the groundwater drainage network report]. *INFN report*. 180 pp (2003).

[55] Catalano PG, Cavinato G, Salvini F, Tozzi M (1986). Analisi strutturale nei Laboratori dell’INFN del Gran Sasso d’Italia. Mem. Soc. Geol. It..

[56] Rovida, A., Locati, M., Camassi, R., Lolli, B. & Gasperini, P. (eds). CPTI15, the 2015 version of the Parametric Catalogue of Italian Earthquakes. Istituto Nazionale di Geofisica e Vulcanologia; doi: http://doi.org/10.6092/INGV.IT-CPTI15 (2016).

[57] Valensise G, Pantosti D (2001). The investigation of potential earthquake sources in peninsular Italy: A review. J. Seismol..

[58] Chiarabba C, Jovane L, Di Stefano R (2005). A new view of Italian seismicity using 20 years of instrumental recordings. Tectonophysics.

[59] De Luca G, Cattaneo M, Monachesi G, Amato A (2009). Seismicity in the Umbria-Marche region integrating the Italian national and regional networks. Tectonophysics.

[60] De Luca G (2000). A detailed analysis of two seismic sequences in Abruzzo, Central Apennines, Italy. J. Seismol..

[61] Frepoli A (2017). Seismic sequences and swarms in the Latium-Abruzzo-Molise Apennines (central Italy): New observations and analysis from a dense monitoring of the recent activity. Tectonophysics.

[62] Bagh S (2007). Background seismicity in the central Apennines of Italy: The Abruzzo region case study. Tectonophysics.

[63] Galli P, Galadini F, Moro M, Giraudi C (2002). New paleoseismological data from the Gran Sasso d’Italia area (central Apennines). Geophys. Res. Lett..

[64] Amoruso A, Crescentini L, Petitta M, Rusi S, Tallini M (2011). Impact of the April 6, 2009 L’Aquila earthquake on groundwater flow in the Gran Sasso carbonate aquifer, Central Italy. Hydrol. Process..

[65] Adinolfi Falcone R (2012). Changes on groundwater flow and hydrochemistry of the Gran Sasso carbonate aquifer due to the 2009 L’Aquila earthquake. Ital. J. Geosci. (Boll. Soc. Geol. It.).

[66] Chiodini G (2011). Geochemical evidence for and characterization of CO_2_ rich gas sources in the epicentral area of the Abruzzo 2009 earthquakes. Earth and Planetary Science Letters.

[67] Cigolini C, Laiolo M, Coppola D (2015). The LVD signals during the early-mid stages of the L’Aquila seismic sequence and the radon signature of some aftershocks of moderate magnitude. J. Environ. Radioactivity.

[68] Plastino W (2010). Uranium groundwater anomalies and L’Aquila earthquake, 6^th^ April 2009 (Italy). J. Environ. Radioactivity.

[69] Barbieri M, Boschetti T, Petitta M, Tallini M (2005). Stable isotopes (^2^H, ^18^O and ^87^Sr/^86^Sr) and hydrochemistry monitoring for groundwater hydrodinamics analysis in a karst aquifer (Gran Sasso, Central Italy). Appl. Geochem..

[70] Petitta M, Tallini M (2002). Idrodinamica sotterranea del massiccio del Gran Sasso (Abruzzo): indagini idrologiche, idrogeologiche e idrochimiche (1994–2001). Boll. Soc. Geol. D’It.

[71] Amoruso A, Crescentini L, Petitta M, Tallini M (2012). Parsimonious recharge/discharge modeling in carbonate fractured aquifers: the groundwater flow in the Gran Sasso aquifer (Central Italy). Journal of Hydrology.

[72] Amoruso A, Crescentini L, Martino S, Petitta M, Tallini M (2014). Correlation between groundwater flow and deformation in the fractured carbonate Gran Sasso aquifer (INFN underground laboratories, Central Italy). Water Resour. Res..

[73] Tallini M, Parisse B, Petitta M, Spizzico M (2013). Long-term spatio-temporal hydrochemical and ^222^Rn tracing to investigate groundwater flow and water-rock interaction in the Gran Sasso (central Italy) carbonate aquifer. Hydrogeology Journal.

[74] Tallini M (2014). Isotope hydrology and geochemical modeling: new insights into recharge process and water-rock interaction in the Gran Sasso fissured carbonate aquifer (Central Italy). Environ. Earth Sci..

[75] Fiorillo F (2015). Long term trend and fluctuations of karst spring discharge in a Mediterranean area (Central-Southern Italy). Environ. Earth Sci..

[76] Scozzafava M, Tallini M (2001). Report: Net infiltration in the Gran Sasso Massif of central Italy using the Thornthwaite water budget and curve-number method. Hydrogeology Journal.

[77] Adinolfi Falcone R, Falgiani A, Petitta M, Tallini M (2006). Characteristics of Gran Sasso INFN laboratory groundwater (inferred from 1996-1998 spot sampling data) to fine-tune the conceptual model of water-rock interaction in carbonate aquifer. Internal Report INFN, LNGS/GEO.

[78] Adinolfi Falcone R (2008). Chemical and isotopic (δ^18^O‰, δ^2^H‰, δ^13^C‰, ^222^Rn) multi-tracing for groundwater conceptual model of carbonate aquifer (Gran Sasso INFN underground laboratory – central Italy). Journal of Hydrology.

[79] D’Agostino N, Chamot-Rooke N, Funiciello R, Jolivet L, Speranza F (1998). The role of pre-existing thrust faults and topography on the styles of extension in the Gran Sasso range (central Italy). Tectonophysics.

[80] De Luca, G., Di Carlo, G. & Tallini, M. Hydraulic pressure variations of groundwater in the Gran Sasso underground laboratory during the Amatrice earthquake of August 24, 2016. Annals of Geophysics 59, Fast Track 5; doi: 10.4401/AG-7200 (2016).

[81] Di Virgilio ADV (2017). GINGER: A feasibility study. Eur. Phys. J. Plus.

[82] Simonelli A (2016). First deep underground observation of rotational signals from an earthquake at teleseismic distance using a large ring laser gyroscope. Annals of Geophysics 59, Fast Track.

[83] Simonelli A (2018). Rotational motions from the 2016, central italy seismic sequence, as observed by an underground ring laser gyroscope. Geophys. J. Int..

[84] Kano Y, Yanagidani T (2006). Broadband hydroseismograms observed by closed borehole wells in the Kamioka mine, central Japan: Response of pore pressure to seismic waves from 0.05 to 2 Hz. J. Geophys. Res..

[85] Jónsson S, Segall P, Pedersen R, Björnsson G (2003). Post-earthquake ground movements correlated to pore-pressure transients. Nature.

[86] Chiodini G (2004). Carbon dioxide Earth degassing and seismogenesis in central and southern Italy. Geophys. Res. Lett..

[87] Doglioni C, Carminati E, Petricca P, Riguzzi F (2015). Normal fault earthquakes or graviquakes. Scientific Reports.

[88] Miller AM (2004). Aftershocks driven by a high-pressure CO_2_ source at depth. Nature.

[89] Crews JB, Cooper CA (2014). Experimental evidence for seismically initiated gas bubble nucleation and growth in groundwater as a mechanism for coseismic borehole water level rise and remotely triggered seismicity. J. Geophys. Res. Solid Earth.

[90] Toutain J-P, Baubron J-C (1999). Gas geochemistry and seismotectonics: a review. Tectonophysics.

[91] King C–Y, Zhang W, Zhang Z (2006). Earthquake-induced groundwater and gas changes. Pure Appl. Geophys..

[92] Di Luccio F (2018). Seismic signature of active intrusions in mountain chains. Sci. Adv..

[93] Girault F (2018). Persistent CO_2_ emissions and hydrothermal unrest following the 2015 earthquake in Nepal. Nat. Commun..

[94] Hotovec-Ellis AJ (2018). Deep fluid pathways beneath Mammoth Mountain, California, illuminated by migrating earthquake swarms. *Sci*. Adv..

